# Public health event communication under the International Health Regulations (2005) in the Western Pacific Region, September 2006-January 2017

**DOI:** 10.5365/2019.10.1.006

**Published:** 2019-09-27

**Authors:** Li Xi, Li Ailan

**Affiliations:** a WHO Health Emergencies Programme, WHO Regional Office for the Western Pacific, Manila, Philippines.

## Abstract

• The International Health Regulations, or IHR (2005), establishes timely communication between the World Health Organization (WHO) and Member States to manage acute public health events and protect health security. Experiences of the WHO IHR contact point for the Western Pacific Region demonstrated the communication mechanism has achieved its functions in the Region.

• Investment in IHR communication as part of the *Asia Pacific Strategy for Emerging Diseases and Public Health Emergencies* (APSED III) during peaceful times between public health emergencies builds capacity, confidence and trust in information sharing during emergencies.

• IHR communication is integral to the national, regional and global epidemic intelligence and risk assessments system.

• Regular simulation exercises (for example, IHR Exercise Crystal) play an important role in testing and strengthening IHR communication.

• IHR communication continues to be vital for Member States and WHO country offices to advise on health security.

The revised International Health Regulations (IHR) (2005), entered into force in June 2007, is a legally binding international agreement on 196 States Parties, including all 194 Member States of World Health Organization (WHO). (*1*) In the Western Pacific Region, National IHR Focal Points (NFPs) have been established in 27 Member States, which are States Parties to the IHR. Communication between WHO and countries through the NFPs is the cornerstone of timely detection of public health risks and effective response to health emergencies. Countries are required to notify WHO of all events that may constitute a public health emergency of international concern (PHEIC) through their NFPs. Strengthening the functions of NFPs is one of the strategic actions to enhance public health emergency preparedness through the implementation of the *Asia Pacific Strategy for Emerging Diseases and Public Health Emergencies* (APSED III). (*2*)

IHR event communication refers to official communication between NFPs and WHO IHR contact points (CPs) at regional and global levels, as well as communication between NFPs in different countries, and between the NFP and relevant departments or agencies within the country to notify public health events; share and verify information; determine whether an event constitutes a PHEIC; and coordinate emergency responses. (*1*) E-mail has been the main mechanism of communication between WHO CPs and NFPs. In addition to e-mail communication, WHO has developed a password-protected web site, the Event Information Site (EIS), to facilitate information sharing with all NFPs. Events posted on EIS, which are often potential PHEICs or public hazards with international impact, are accessible to all NFPs. (*3*)

IHR Exercise Crystal, a simulation exercise organized by the WHO Western Pacific Regional Office (WPRO) to test and strengthen event communication between the IHR CP of WPRO (WPRO IHR CP) and NFPs in the Region, has been conducted in the Western Pacific Region annually since 2008, (*4*) except that in 2009 NFPs communicated frequently with the WPRO IHR CP during the PHEIC of pandemic influenza A(H1N1). The role of IHR Exercise Crystal in testing and strengthening the communication functions is well recognized in the 10-year evaluation of APSED and meetings of the Technical Advisory Group on APSED. (*5*, *6*)

This regional analysis presents an evaluation of the extent and function of IHR event communication in the WHO Western Pacific Region as informed by e-mail records of the WPRO IHR CP and experiences from IHR Exercises Crystal. Specifically, we classified each event under one IHR article related to communications from States Parties to WHO and analysed the number and types of events communicated under the relevant IHR articles: Article 6 Notification; Article 8 Consultation; Article 9 Other reports; Article 10 Verification; Article 44 Collaboration and assistance. (*1*) We also summarized the types of events posted on EIS and the scopes, objectives and results of IHR Exercise Crystal from 2008 to 2016.

## IHR communication

E-mail was the main mechanism of communication between the WPRO IHR CP and NFPs. In rare cases, documents were faxed to WPRO, and the WPRO IHR CP received e-mail notices when faxes arrived. Telephone calls were infrequent and were always accompanied by an e-mail.

E-mails were retrieved from the archives of the mailbox of the WPRO IHR CP. The e-mails covered communications from September 2006, when the mailbox was put into use, to January 2017 at the time of analysis. The contents of e-mails received from NFPs and external partners or from other regional WHO IHR CPs were reviewed to determine the disease or public health hazard reported, the IHR article under which the event was communicated, the countries involved and the time of the communication. In case of novel influenza viruses, the notification of the first case in a Member State is counted as one event, while the subsequent reports of additional cases were considered as updates to the event. Event information disseminated by the International Food Safety Authorities Network (INFOSAN), in which NFPs were copied, were not included in the analysis. E-mail exchanges among WHO staff other than those between the designated WHO IHR CPs were not included; these were considered to be internal business processes after events were communicated to WHO.

**Figure 1:**
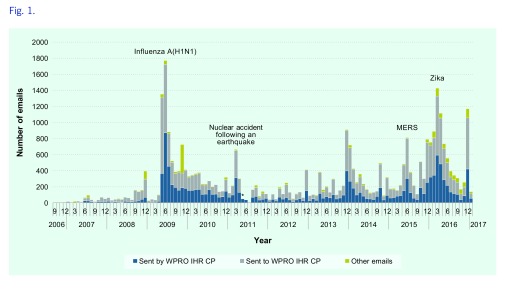
**Number of emails by months in the mailbox of the WPRO IHR CP, September 2016–January 2017**

After removing duplicates, a total of 34 438 e-mails were recovered from the archives of WPRO IHR mailbox from 11 September 2006 to 12 January 2017, of which 2944 (8.5%) were IHR exercise messages. E-mails received from 1 May to 25 August 2011 could not be recovered due to archiving issues. Among the 34 438 retrieved e-mails, 13 252 (38%) were sent from the WPRO IHR CP; and 18 922 (55%) were sent to the WPRO IHR CP, including 5523 e-mails that copied the WPRO IHR CP. The other 2264 e-mails (7%) included fax notices, surveillance reports that did not list the recipients and e-mails sent to a very large group of recipients for which we could not determine if WPRO was on the direct or copying lines as the lines were truncated when data were imported into an Access database. **Fig. 1** shows the number of e-mails by month.

[**insert** Figure 1]

**Figure 2:**
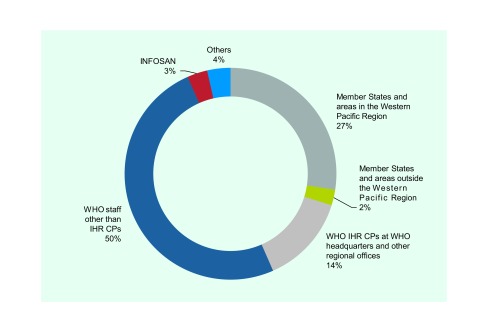
**Number of emails received by the WPRO IHR CP by sender categories, September 2016–January 2017**

Of the 21 186 e-mails received by the WPRO IHR CP, 5809 (27.4%) were from Member States in the Western Pacific Region, 508 (2.4%) were from Member States and areas outside the Western Pacific Region, 2881 (13.6%) were from WHO IHR CPs in WHO headquarters or other WHO regional offices, 10 582 (49.9%) were from WHO staff other than WHO IHR CPs, 612 (2.9%) were from INFOSAN and 41 (0.2%) were from international partner organizations (**Fig. 2**). The remaining 753 (3.6%) included autoreplies, subscriptions to event alerts and system-generated e-mails. All 27 NFPs in the Western Pacific Region communicated with the WPRO IHR CP. Thirty-four Member States and areas^§^ of the WHO Western Pacific Region, including those that are not States Parties to the IHR, communicated with the WPRO IHR CP. Thirty-five countries outside the Western Pacific Region communicated with the WPRO IHR CP.

[**insert** Figure 2]

### Notification (IHR Article 6, Article 9)

A total of 89 notifications of potential PHEIC under Article 6 were received from Member States and areas of the Western Pacific Region as of 12 January 2017 (France and the United States of America made notifications on behalf of their territories in the Pacific). All States Parties in the Western Pacific Region, except for Tuvalu, made at least one notification of a potential PHEIC to the WPRO IHR CP during this period. Thirty-three diseases and one nuclear accident following an earthquake were reported to the WPRO IHR CP. Forty-three (48%) notifications were about novel influenza viruses, including 32 notifications on pandemic H1N1, four on H5N1, two on H7N9, two on H9N2 and one each for H10N8, H3N2, and H5N6. Eleven notifications were about Zika virus disease, including microcephaly and Guillain–Barré syndrome associated with Zika virus disease.

In addition to notifications under Article 6, five notifications cited Article 9, which asks States Parties to inform WHO of a public health risk identified outside their territory that may cause international disease spread. These notifications included cases of Zika virus disease and cholera imported from other countries, a norovirus outbreak during an international gathering, and a close contact of a Middle East respiratory syndrome (MERS) case who travelled internationally.

### Information sharing and consultation with WPRO (IHR Article 8)

Five countries consulted with the WPRO IHR CP about 14 events that either did not require notification as a potential PHEIC or did not have enough information to determine if PHEIC criteria had been met. None of these events was declared as a PHEIC. In addition, 12 NFPs shared information with the WPRO IHR CP about 27 diseases or disasters that might have international impact but did not constitute a PHEIC.

### Verification (IHR Article 10)

The WPRO IHR CP made 13 requests for verification of events known to WHO from sources other than notifications and consultations. Of these, eight events (62%) had evidence of response from NFPs within 24 hours.

### Intercountry collaboration and assistance (IHR Article 44)

The IHR has been widely used by NFPs for communication between countries. The WPRO IHR CP facilitated or was copied in communication between NFPs in 273 events. In 237 events, NFPs initiated the communication to provide information to other NFPs, including contact tracing in 71 events, follow-up for patient management in 10 events, reporting travellers or foreign nationals under public health observation/investigation in 17 events and sharing information of imported or exported cases of communicable diseases in 135 events. The most frequently reported diseases were tuberculosis (53 events), measles (29 events), chlamydia (16 events), Legionnaire’s disease (15 events), MERS (14 events), Zika virus disease (12 events) and Ebola virus disease (10 events for sharing notice of travellers under monitoring with low risk exposure and two events for contact tracing). Due to the pandemic nature of H1N1, communications following initial notifications in each country related to contact tracing of H1N1 cases and antiviral resistance were not counted as separate from initial notification.

In 36 instances, NFPs made requests for information from another NFP. These communications usually took place when NFPs wanted to verify media reports of diseases in another country or ask questions following an EIS posting.

### Information sharing through EIS

**Figure 3:**
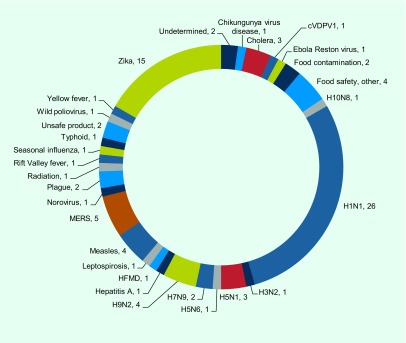
**Types and counts of public health events posted on the Event Information Site from the Western Pacific Region, 2016–January 2017**

Events that are potential PHEICs or other health hazards with international impact are posted on EIS following notifications or other communications under the IHR. NFPs of all States Parties can view the event information on the password-protected web site. A total of 90 postings from 24 countries or areas in the Western Pacific Region were shared on EIS as of January 2017. Thirty-nine (43%) of the postings were about influenza. **Fig. 3** shows the type of public health events from the Western Pacific Region posted on EIS between 2007 and 2016.

Avian influenza A(H7N9) in China had the highest number of updates for a single event in the Western Pacific Region. The virus was first laboratory-confirmed in China on 31 March 2013 and notified to WHO on 1 April 2013. The first posting on EIS was published on 1 April 2013 and was accessible to all NFPs. The additional cases were reported daily during the first season of the epidemic, and weekly or monthly in the subsequent seasons. The reporting frequency increased during the seasons when the number of H7N9 cases increased. Between 1 April 2013 and 12 January 2017, 166 updates of H7N9 were posted on EIS. Sixty-two out of 177 updates were posted on the same day. The median time from reporting to EIS posting was 1.62 days.

[**insert** Figure 3]

### Requesting information from WHO

Forty-two requests for further information were sent from NFPs to the WPRO IHR CP, often following media reports or EIS postings of events in another country.

## IHR Exercise Crystal

IHR Exercise Crystal has been held annually from 2008 to 2016, with the exception of 2009 when the real-world event of pandemic influenza A(H1N1) tested IHR communication between countries and WHO. The scope of IHR Exercise Crystal has been evolving with increased complexity (**Table 1**). The main objectives of IHR Exercise Crystal have been consistent over the years: to strengthen the accessibility of NFP contact details, event notification process and information sharing through developing postings for EIS. Additional objectives have been added with more functions tested (**Table 2**).

**Figure 4:**
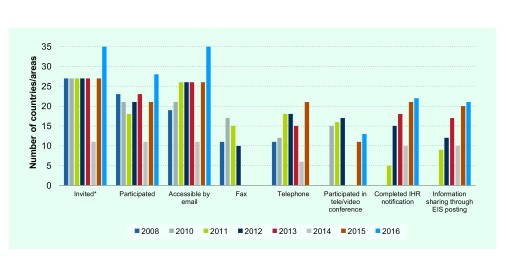
**Member States' participation and performace in IHR Exercise Crystal, 2008–2016**

All 27 countries in the Region were invited to participate in IHR Exercise Crystal, except in 2014, when 11 countries were invited to participate in a joint IHR-INFOSAN exercise. In 2016, eight territories and areas in the Region were invited in addition to the 27 countries (**Fig. 4**). The accessibility of NFPs by e-mail increased steadily over 2008–2016. In 2008, 70% (19 out of 27) of NFPs were accessible by e-mails. This percentage increased to over 95% since 2011. The other ways of communication, including fax, telephone, teleconference and text messaging were tested in some years with varying results. The number of NFPs who completed the expected tasks increased over the years. In 2015, 21 NFPs made notifications during the allotted exercise time, an increase from five NFPs in 2011. In 2015, 20 NFPs completed the draft EIS posting in the allotted exercise time compared to eight NFPs in 2011.

Feedback was collected from NFPs following the exercises. NFPs have commented that the scopes were appropriate and the objectives were achieved; the exercises “enhanced collaboration with partners and promoted teamwork”; and the exercises “strengthened IHR event-related communication” between NFPs and WHO. NFPs recommended that the exercise be continued.

**Table 1:** Scopes of IHR Exercise Crystal, 2008–2016

Year	Event
2008	Verification of an outbreak of unknown etiology occurring in the participating country.
2010	Notification of a Public Health Emergency of International Concern (PHEIC) and share the information on Event Information Site (EIS).
2011	Notification of a potential PHEIC (severe acute respiratory infection of unknown etiology) and share the information on EIS.
2012	Notification of a potential PHEIC (influenza-like illness) and share the information on EIS.
2013	Notification of a potential PHEIC (severe acute respiratory infection of unknown etiology) and share the information on EIS.
2014*	Joint exercise between NFPs and INFOSAN emergency contact points on notification and information sharing of an outbreak of Verocytotoxin-producing *Escherichia coli* infection caused by an internationally distributed food product.
2015	Notification of a novel avian influenza virus and consulting and conferring with WHO about potential impacts on travel and trade.
2016	Notification of a disease of unknown etiology, communication with national disaster management and providing information for IHR Emergency Committee.

* An Ebola simulation exercise was conducted in addition to the regular IHR Exercise Crystal.

**Table 2:** Objectives of IHR Exercise Crystal, 2008–2016

Objectives	2008	2010	2011	2012	2013	2014	2015	2016
Validate the accessibility of NFPs using registered contact details	Yes	Yes	Yes	Yes	Yes	Yes	Yes	Yes
IHR notification process	No	Yes	Yes	Yes	Yes	Yes	Yes	Yes
Draft a posting to share information through the IHR Event Information Site	No	Yes	Yes	Yes	Yes	Yes	Yes	Yes
Test the use of teleconferencing	No	Yes	Yes	Yes	No	No	Yes	Yes
Additional objectives (see footnotes)	a	b	-	-	c	d	e	f

a. Validate IHR verification process; test the WHO guide on IHR communication and Duty Officer System.

b. Test the WHO guide on IHR communication and Duty Officer System.

c. Improve the engagement of WHO country offices in facilitating communication.

d. Validate the accessibility of INFOSAN emergency contact points; facilitate communication and collaboration between them during a foodborne disease emergency event.

e. Practise and evaluate the NFP understanding and use of the IHR principles and obligations regarding travel restrictions and border measures.

f. Examine protocols when working with non-health actors, particularly national disaster management agencies; familiarize participants with the IHR Emergency Committee.

In October 2014, in response to the global Ebola virus disease (EVD) epidemic, an Ebola simulation exercise and an Ebola preparedness survey were conducted in addition to the regular IHR Exercise Crystal. (*7*) Twenty-three countries participated in the exercise that simulated the scenario of an imported case of EVD. The majority of the countries were able to complete the expected actions, including sharing national EVD guidelines and response plans, providing technical advice on contact tracing, case management and patient transportation and drafting a press release. The exercise identified specimen referral as an area for improvement.

[**insert** Figure 4]

## Discussion

Ten years after IHR (2005) entered into force, the communication mechanism set up by IHR (2005) has been functional in supporting Member States to report potential public health risks to WHO and other countries. All States Parties in the Western Pacific Region have made contact with the WPRO IHR CP, and all but one made a notification of potential PHEICs to WPRO. IHR event communication has also been used for sharing information with WHO on events that do not constitute a PHEIC. WPRO has used IHR event communication to verify media and other reports with NFPs. Countries that are actively screening media or other information sources for public health risks used IHR event communication to verify information from WHO or another country. The network has become an important information source for risk assessment to both WHO and the countries. It is an integral component of global epidemic intelligence system.

The IHR communication mechanism has been widely used by NFPs for intercountry collaboration and assistance. The majority of IHR communication occurred between countries for information sharing, contact tracing and follow-up of patients to ensure continuity of infectious disease management.

IHR communication has an all-hazards approach. While most of the events reported through IHR communication were infectious diseases, other public health concerns, including natural disasters, nuclear accidents and food safety issues were reported through IHR.

Timely communication during epidemics (the H1N1 pandemic influenza, Zika virus disease, MERS) exemplifies the importance of investment in public health preparedness in peaceful times between major public health emergencies to build confidence and trust between WHO and Member States in information sharing. The capacity of NFPs has been strengthened in the past 10 years, which can be observed through the simulation exercises: increasing numbers of NFPs could complete the tasks of making notifications and developing an EIS posting in the exercises. IHR Exercise Crystal is being replicated globally as a model to test and improve the functions of IHR communication.

The analysis had several limitations. First, multiple IHR articles may apply to the same event, and countries reported events of similar nature to WHO citing different IHR articles. We classified the events under each IHR article based on our best understanding of the content and context, while acknowledging the classification might be subjective in some events and we didn’t attempt to analyse how many times articles in other parts of IHR (for example, Part IV Points of entry, Part V Public health measures) have been applied. Second, only e-mails were analysed. Other means of IHR event communication have been used, for example telephones and fax, although it is rare that events are reported without any e-mail record. Additionally, e-mails received from May to July 2010 could not be retrieved, and e-mails sent by the WPRO IHR CP were not systematically archived. The WPRO IHR CP could potentially improve its information management by developing a system to routinely archive messages. Third, we likely underestimated the number of verifications from WHO. In countries with WHO country offices, the requests for verifying media reports and other reports were often communicated through WHO country offices, which then facilitated communication with the in-country counterparts. These communications may not have involved direct communication between the WPRO IHR CP and NFPs, and therefore were not covered by this analysis. Fourth, we also likely underestimated the number of intercountry communications as the WPRO IHR CP was not always copied in communications between NFPs. Given the communications not covered in the analysis, this report presents a conservative picture of the extent of IHR communication within the Region.

In conclusion, IHR communication has played a pivotal role in communicating PHEIC and other public health risks between countries and WHO and among countries. IHR Exercise Crystal played a positive role in strengthening IHR communication and collaboration. The capacity of NFPs improved as shown in IHR Exercise Crystal.

Timely IHR event communication between NFPs and WHO is an integral component of the global and regional surveillance and risk assessment system that protects national, regional and global health security. With the establishment and implementation of the WHO new Health Emergencies Programme, (*8*) it is expected that the functions of the NFPs and the WHO IHR CPs will be further strengthened and advanced. The experiences and lessons from the Western Pacific Region could be a useful contribution to the achievement of the mission of the global programme to strengthen the capacity to prevent, detect and respond to public health threats worldwide.

### Ethics statement

Ethical review is not required since reviewing the information communicated through the IHR mechanism is a public health practice activity and the purpose of the analysis is to evaluate and improve the IHR communication mechanism.

## References

[1] International Health Regulations. (2005). Geneva: World Health Organization; 2008 (https://www.who.int/ihr/publications/9789241580496/en/)

[2] Asia Pacific strategy for emerging diseases and public health emergencies (APSED III): advancing implementation of the International Health Regulations (2005). Manila: WHO Regional Office for the Western Pacific; 2017 (https://iris.wpro.who.int/handle/10665.1/13654)

[3] WHO event management for international public health security: Operational procedures. Geneva: World Health Organization; 2008 (https://www.who.int/ihr/publications/WHO_HSE_EPR_ARO_2008_1/en/)

[4] Exercise Crystal IHR. (2015). Manila: WHO Regional Office for the Western Pacific; 2015 (https://apps.who.int/iris/handle/10665/246428)

[5] Asia Pacific strategy for emerging diseases: evaluation report 2005–2015. Manila: WHO Regional Office for the Western Pacific; 2018 (https://iris.wpro.who.int/handle/10665.1/14028)

[6] Meeting of the Technical Advisory Group on the Asia Pacific Strategy for Emerging Diseases and Public Health Emergencies (APSED III). Manila, Philippines, 11–13 July 2017: meeting report. Manila: WHO Regional Office for the Western Pacific; 2017 (https://iris.wpro.who.int/handle/10665.1/13980)

[7] Xu Z, Pavlin B, Squires RC, Chinnayah T, Konings F, Lee CK, et al. World Health Organization Regional Office for the Western Pacific Ebola Emergency Support Team. Ebola preparedness in the Western Pacific Region, 2014. West Pac Surveill Response. 2015 1 26;6(1):66–72. 10.5365/wpsar.2014.5.4.004 25960926 PMC4410100

[8] WHO Health Emergencies Programme: progress and priorities, Financing dialogue 31 October 2016. Geneva: World Health Organization; 2016 (https://www.who.int/about/finances-accountability/funding/financing-dialogue/whe-update.pdf)

